# Transcriptional Regulation of Ascorbic Acid During Fruit Ripening in Pepper (*Capsicum annuum*) Varieties with Low and High Antioxidants Content

**DOI:** 10.3390/plants8070206

**Published:** 2019-07-04

**Authors:** Pasquale Chiaiese, Giandomenico Corrado, Maria Minutolo, Amalia Barone, Angela Errico

**Affiliations:** Dipartimento di Agraria, Università degli Studi di Napoli “Federico II”, via Università 100, 80055 Portici (NA), Italy

**Keywords:** antioxidants, vitamin C, gene transcription, germplasm, sweet pepper

## Abstract

Research on plant antioxidants, such as ascorbic acid (AsA) and polyphenols, is of increasing interest in plant science because of the health benefits and preventive role in chronic diseases of these natural compounds. Pepper (*Capiscum annuum* L.) is a major dietary source of antioxidants, especially AsA. Although considerable advance has been made, our understanding of AsA biosynthesis and its regulation in higher plants is not yet exhaustive. For instance, while it is accepted that AsA content in cells is regulated at different levels (e.g., transcriptional and post-transcriptional), their relative prominence is not fully understood. In this work, we identified and studied two pepper varieties with low and high levels of AsA to shed light on the transcriptional mechanisms that can account for the observed phenotypes. We quantified AsA and polyphenols in leaves and during fruit maturation, and concurrently, we analyzed the transcription of 14 genes involved in AsA biosynthesis, degradation, and recycling. The differential transcriptional analysis indicated that the higher expression of genes involved in AsA accumulation is a likely explanation for the observed differences in fruits. This was also supported by the identification of gene-metabolite relations, which deserve further investigation. Our results provide new insights into AsA differential accumulation in pepper varieties and highlight the phenotypic diversity in local germplasm, a knowledge that may ultimately contribute to the increased level of health-related phytochemicals.

## 1. Introduction

In recent decades, the link between nutrition and health has gained universal acceptance, and therefore, increasing importance has been given to dietary regimes based on vegetables rich in antioxidants [1]. The health-promoting properties of antioxidants correlate to the ability to counteract the effect of oxidative stress and the over-production of free radicals in cells, thus providing protection against aging and several degenerative cellular conditions [2].

The pepper (*Capsicum annuum* L.) is the most economically important species of its genus, widely recognized as a valuable source of bioactive compounds [3,4]. Sweet peppers have long been considered a main dietary source of antioxidants, such as vitamin C (ascorbic acid, AsA) [4]. AsA is the primary water-soluble antioxidant in plants, and it is also implicated in plant development and hormone signaling [3,5]. Peppers are also rich in polyphenols, another important class of antioxidants that can provide several health benefits [6]. Phenolic compounds are a large group of secondary metabolites whose accumulation in plants is often linked to stress response [1,7]. The amount and type of polyphenols in edible products can have a strong effect on taste (e.g., determining bitterness and astringency) and on the appearance of the product (i.e. color) [8].

The level of bioactive compounds in pepper, as well as in other horticultural products, is affected by the maturity stage of the fruit [9,10,11]. Peppers are harvested and consumed at different ripening stages, from immature green to fully ripe. Variations occurring during maturation do not only have well-known agronomical implications (e.g., taste, color, size, post-harvesting properties, etc.), but are relevant to determine fruit quality and use too. Differences in AsA accumulation have also a genetic base. Different studies indicated that the accumulation of AsA in fruits may vary among pepper cultivars [10,12,13], although not all aspects of this phenomenon are fully elucidated (e.g., correlation of AsA content with other organs and prevalence of the different biosynthetic pathways, also at different stages) [14,15]. 

AsA accumulation in plants is a finely tuned process that results from the balance of de novo biosynthesis, recycling, and degradation [16,17]. In addition, AsA accumulation also depends on the relation between different source- and sink-organs [18]. The de novo synthesis of AsA in higher plants relies mainly on the L-galactose pathway, also known as the Smirnoff–Wheeler pathway [19], which, in a number of steps, converts simple sugars into galactose and then into ascorbic acid. Additional pathways for AsA biosynthesis are those based on the conversion of 1-D-myo-inositol, D-galacturonic acid or L-gulose, although the L-gulose pathway is also considered a branch of the L-galactose pathway [20]. Besides synthesis, the pool of AsA in cells is also maintained by recycling [16,21]. In a simplistic way, AsA is oxidised by reactive oxygen species (ROS) into monodehydroascorbate (MDHA), a short-lived intermediate that can be converted into dehydroascorbate (DHA) or to ascorbate. Genes involved in AsA biosynthesis, recycling and degradation have been cloned and characterized in different horticultural species [22,23]. While some genes have been highlighted as important determinants of AsA content, the molecular mechanisms that regulate AsA accumulation and dynamics remain not entirely clarified. It is generally accepted that L-galactose pathway has the largest influence on AsA metabolism [23,24]. Nonetheless, the genetic regulation as well as the importance of the expression of the AsA-biosynthetic genes are not well specified, also because AsA-dependent negative feedback are involved in the regulation of the biosynthesis [22,25].

The study of the accumulation of main antioxidant compounds during maturation is getting increasing interest in plant science because their content is considered an important parameter concerning the quality of vegetables [26,27]. The bio-fortification for vitamin C and other antioxidants in horticultural crops remains a challenge for plant science [28,29], making the identification of genotypes with an increased level of bioactive compounds valuable. The aim of this work was to investigate the molecular basis of AsA accumulation at the transcriptional level in two pepper varieties with contrasting phenotype. We quantified AsA and polyphenol content in leaves and during fruit maturation, in order to understand the dynamics of the compound accumulation. We also studied the expression levels of genes involved in both synthesis and recycling of the AsA to have an integrative view on the factors that may contribute to different AsA accumulation in pepper fruits. 

## 2. Results

### 2.1. AsA Concentration in Leaves and during Fruit Ripening

**Figure 1:**
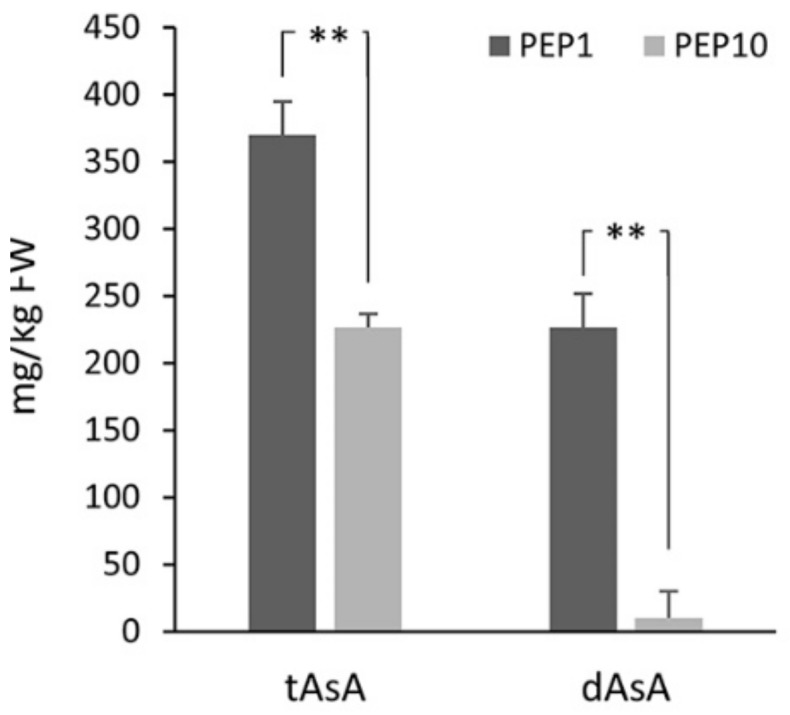
Total ascorbic acid (tAsA) and reduced ascorbic acid (dAsA) concentration (mg/kg FW) in leaves of the two pepper varieties. Data are mean ± s.d. of three biological replicates. Asterisks indicate a statistically significant difference between varieties (** *p* < 0.01; *t*-test).

The concentration of total ascorbic acid (tAsA) and reduced AsA (dAsA) in leaves of the two cultivars differed significantly. Specifically, the variety PEP1 had a significantly higher content of tAsA and dAsA compared to PEP10 (Figure 1). Moreover, differences were also evident in the percentage of reduced ascorbate relative tAsA, which was 61% for PEP1 and 4% for PEP10. 

**Figure 2:**
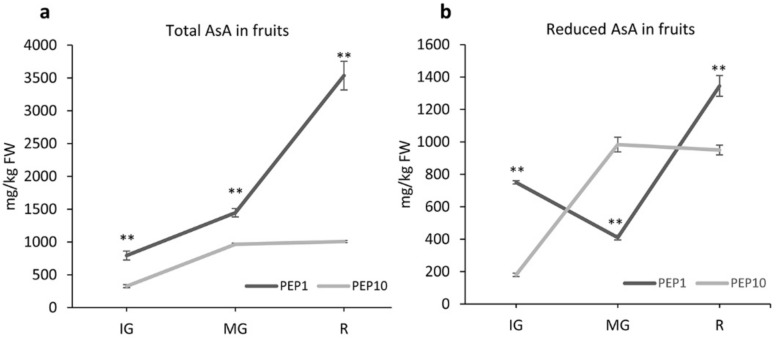
Total ascorbic acid (tAsA) (**a**) and reduced ascorbic acid (dAsA) (**b**) concentration (mg/kg FW) during fruit development. Data are mean ± s.d. of three biological replicates. IG: immature green; MG: mature green; R: red. At each stage, asterisks indicate a statistically significant difference between varieties (** *p* < 0.01; *t*-test).

For the analysis of AsA in fruits, peppers were harvested at identical phenological stages throughout the course of the experiment, mainly considering the development (e.g., color and size) (Supplementary Materials, Figure S1). The concentrations of tAsA and dASA varied significantly between the two cultivars and in relation to the ripening stage (Figure 2).

While at the different stages under investigation (i.e., immature green, IG, mature green, MG, and mature, fully red, R) PEP1 always accumulated more tASA than PEP10, the difference in total ascorbic acid between the two varieties increased during fruit development. PEP11 accumulated more tAsA following MG, where fruits reached their final size, while it did not significantly increase in PEP 10. In this variety, a similar trend was also present for the dAsA, whose concentration did not significantly change at end of fruit cycle. While the accumulation of tASA and dAsA during maturation followed the same trend in PEP10, in PEP1 the increase of tAsA was accompanied by a decrease in dAsA and vice versa. 

This is also illustrated by the proportion of reduced AsA present in the total AsA, a broad indicator of the cellular oxidative stress, which showed an opposite trend in the two varieties (Supplementary Materials, Figure S2). In PEP1 this ratio declined from IG to MG, while it increased in PEP10. The difference between the two varieties slightly varied from MG to R stage. 

Collectively, the data indicated that the higher accumulation of AsA in the PEP1 variety is not organ specific, being also evident in leaves, and that the two varieties differ not only in the concentration, but also in the accumulation trend of AsA during fruit development and maturation. The largest difference was present at the mature stage, with PEP1 accumulating 3.5x tAsA than PEP10.

### 2.2. Polyphenol Concentration in Leaves and during Fruit Ripening

**Figure 3:**
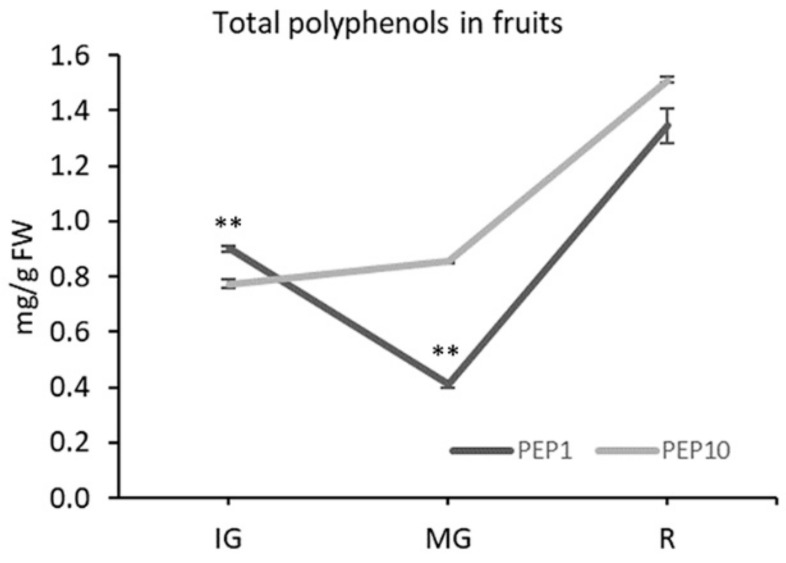
Total polyphenol concentration (mg/g FW) during fruit development. Data are mean ± s.d. of three biological replicates. IG: immature green; MG: mature green; R: red. At each stage, asterisks indicate a statistically significant difference between varieties (** *p* < 0.01; *t*-test).

In leaves, the total polyphenol (TP) content of the PEP1 variety (1.4 ± 0.05 mg/g FW) was higher than the PEP10 variety (0.93 ± 0.01 mg/g FW). For the analysis of the TP concentration in fruits, peppers were harvested at three phenological stages, as for the AsA experiments. In fruits, the TP content varied in each cultivar during fruit development (Figure 3).

At immature green, the PEP1 variety had the higher amount of TP, as in leaves. Although limited to three stages, in this variety, the accumulation trend of TP highly correlated to the AsA accumulation during fruit maturation (R^2^ = 0.98), reaching the minimum value at MG. The TP content in PEP10 increased during fruit development, reaching the maximum value at R. At this stage, however, differences between PEP10 and PEP1 were not significant.

### 2.3. Relative Gene Expression of AsA-related Candidate Genes in Pepper

Considering the significant differences in AsA concentration, we monitored changes in the expression level of genes involved in the AsA biosynthesis in leaves and during fruit ripening. The comparative analysis was performed considering the PEP10 variety as control genotype, i.e., the gene expression in the PEP1 is relative to that of PEP10, arbitrarily set as 1. Briefly, we analyzed 8 genes involved in the AsA biosynthetic pathways (GDP-mannose pyrophosphorylase1 (GMP1), GDP-mannose pyrophosphorylase2 (GMP2), GDP-mannose-3′-5′-epimerase (GME), GDP-l-galactose transferase (GGP/VTC2), L-galactose-1-phosphate phosphatase (GPP/VTC4), L-galactono-1,4-lactone dehydrogenase (GalLDH), myo-inositol oxidase (MIOX), and aldo-keto reductase (AKR)) and 6 genes involved in degradation and recycling of AsA (ascorbate oxidase (AO), ascorbate peroxidase1 (APX1), ascorbate peroxidase2 (APX2), ascorbate peroxidase3 (APX3), monodehydroascorbate reductase1 (MDHAR1) and monodehydroascorbate reductase2 (MDHAR2)). We used the beta-tubulin housekeeping gene as an endogenous reference for the normalization of the expression levels of the target genes.

**Figure 4:**
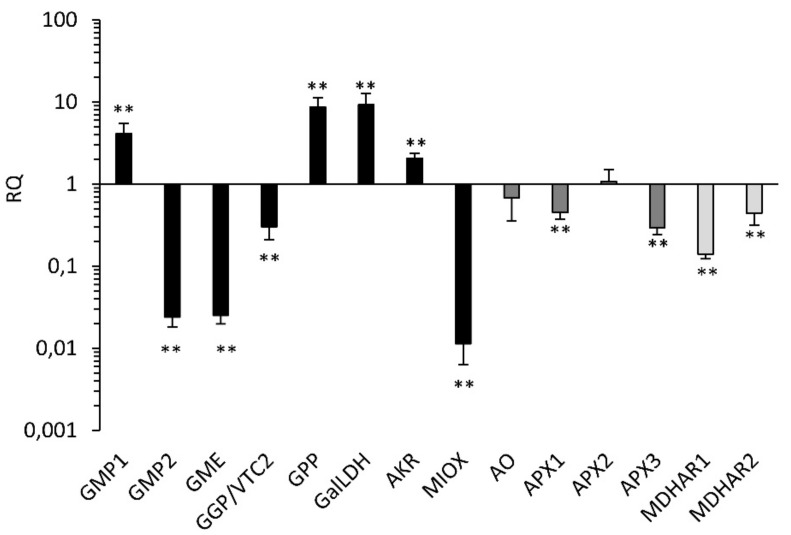
Relative gene expression by real-time PCR of genes involved in AsA accumulation in leaves of the two pepper varieties (black bars designate genes involved in AsA biosynthesis, dark grey bars those involved in AsA oxidation, and light grey bars those involved in recycling, according to Alos et al. 2013). Quantities (RQ) are relative to the calibrator genotype (PEP10) and are graphed on a logarithmic scale. For each gene, asterisks indicate that the 2^−ΔCt^ values were significantly different between the test and calibrator genotype (** *p* < 0.01; *t*-test).

The gene expression analysis in leaves indicated the presence of significant differences between the two varieties, with four over-expressed genes in the PEP1 (namely, GMP1, GPP, GalLDH, and AKR) that are all involved in the biosynthetic pathways that lead to the production of L-Galactono-1,4-lactone (Figure 4). 

On the other hand, the MIOX gene was the most highly under-expressed, suggesting that, at least in this variety, the L-Ascorbic acid biosynthesis from the alternative myo-inositol pathway contributes little to the higher accumulation of ascorbic acid. Among the genes involved in ascorbic acid recycling, the two MDHARs and two APXs coding for enzymes putatively located in the cellular organelles were downregulated in PEP1. On the contrary, the APX2 gene, whose product is predicted to be located in the cytosol, was not differentially expressed. Overall, the gene expression analysis indicated that in the highly accumulating AsA variety (PEP1), ascorbate recycling is reduced. 

**Figure 5:**
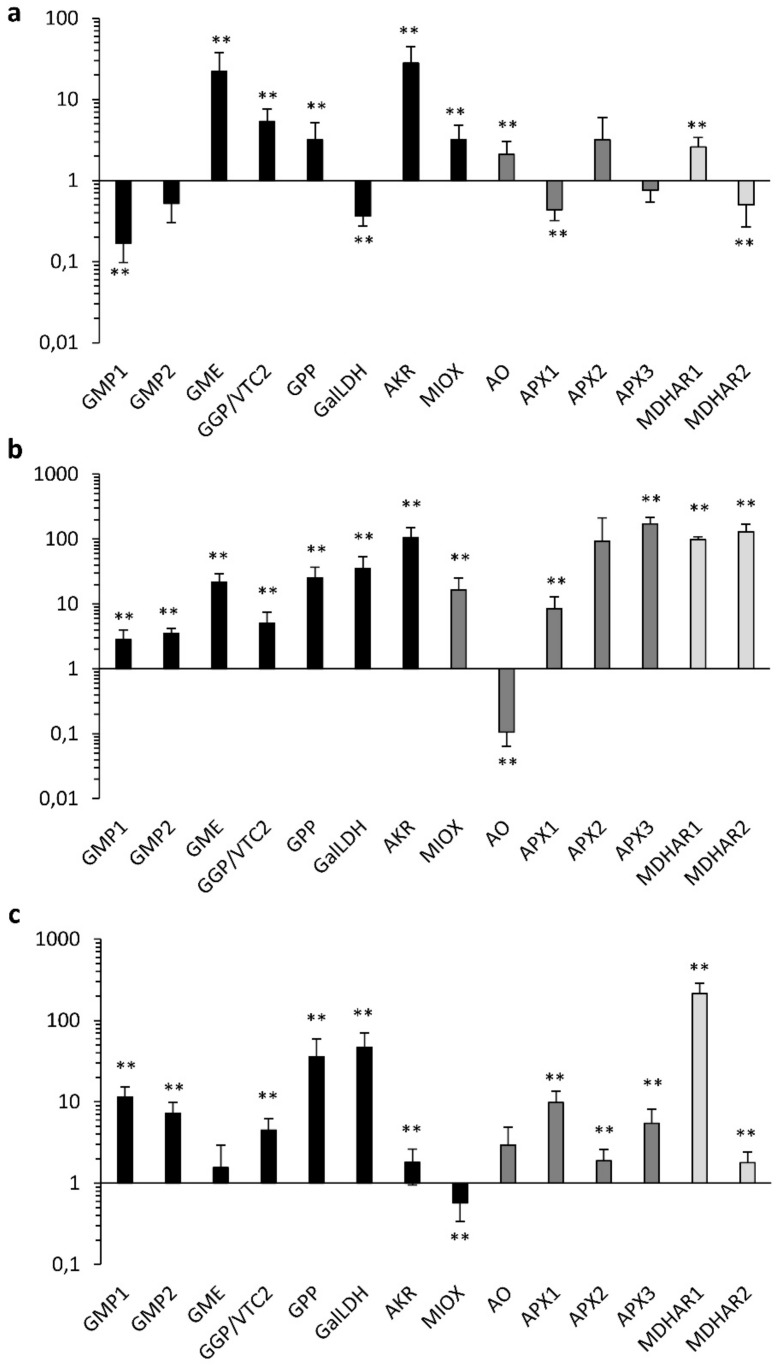
Relative gene expression by real-time PCR of genes involved in AsA accumulation in fruits of the two pepper varieties (black bars designate genes involved in AsA biosynthesis, dark grey bars those involved in AsA oxidation, and light grey bars those involved in recycling, according to Alos et al. 2013). Quantities (RQ) are relative to the calibrator genotype (PEP10) and are graphed on a logarithmic scale. (**a**): immature green (IG); (**b**): mature green (MG); (**c**): red (R). For each gene, asterisks indicate that the 2^−ΔCt^ values were significantly different between the test and calibrator genotype (** *p* < 0.01; *t*-test).

For the analysis of the gene expression during fruit maturation, we compared the two varieties at three stages, IG, MG, and R (Figure 5).

The expression pattern in leaves and in fruits was different, further confirming that the ascorbic acid biosynthesis is differentially regulated in different plant organs [30]. Taking into consideration the different stages, seven genes were statistically overexpressed in the PEP1 fruit at immature green, with five genes involved in AsA accumulation (GME, GGP/VTC2, GPP, MIOX, and AKR) and two involved in AsA recycling/oxidation (AO, and MDHAR1). Moreover, IG was the only stage in which GMP1 and GMP2 were not upregulated. Consistent with the observed accumulation dynamics of tAsA, 12 (respectively, 11) genes were upregulated in PEP1 compared to PEP10 at MG (respectively, R). Although the comparison among different maturation stages may be affected by variation in the expression level of the reference gene, it is also evident that the level of overexpression at MG and R was higher compared to IG. In absolute terms, the MG was characterized by the strongest overexpression of several genes (all but AO). This is likely to be needed to support the increasing difference in AsA accumulation between PEP1 and PEP 10 that was recorded during the MG to R transition. During fruit maturation, the expression level of genes involved in the additional pathways for AsA biosynthesis (i.e. MIOX in the myo-inositol pathway, and AKR, in the galacturonic acid pathway) followed the same pattern. Specifically, these genes were overexpressed at IG, peaked at MG, and then declined at R. This suggests that the non-canonical pathways for the de novo synthesis of AsA also significantly contribute to the higher AsA accumulation in PEP1. 

**Figure 6:**
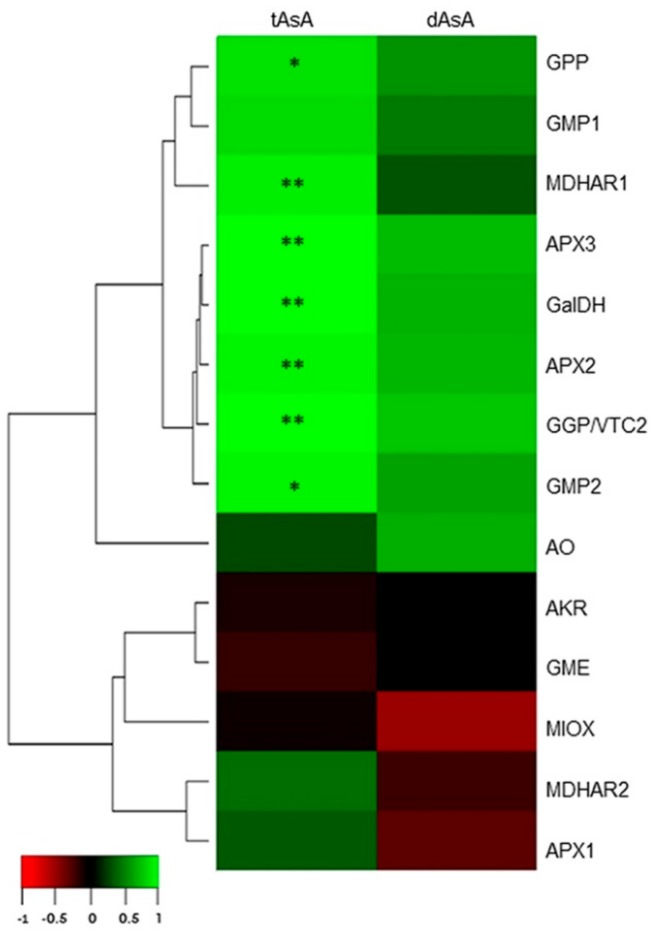
Heatmap of the correlations between gene expression states and tAsA or dAsA in pepper fruits. Gradation from red to green represents negative from positive correlations according to the color scale at the bottom. Asterisks indicate a statistical significant correlation (*: *p* < 0.05; **: *p* < 0.01).

We employed a numerical approach to integrate gene expression and metabolic data [31]. Specifically, we analyzed the correlation between gene expression states and tAsA or dAsA during fruit development. Significant positive correlations were present for half of the genes considering the total AsA (Figure 6). 

Genes associated to the L-galactose pathway highly correlated with tAsA, while those related to the additional AsA biosynthetic routes did not display either a significant or a strong correlation with metabolites. Moreover, the expression of two genes related to L-Ascorbic acid oxidation and recycling positively correlated with tAsA content. Overall, the data indicated, in pepper fruits, the occurrence of functionally related genes that show a coherent association with their expected metabolites, which is consistent with the higher level of expression in the AsA-accumulating variety.

## 3. Discussion

Plant-food is the main dietary source of AsA and polyphenols for humans. For this reason, increasing importance and value is currently given to horticultural products with high content of antioxidants [32]. The identification of varieties with higher level of health-promoting compounds provides viable opportunities not only to offer high-quality products, but also to improve our understanding of the complex dynamics that underlies the accumulation of these beneficial compounds [33]. A preliminary study on 18 local varieties identified two varieties with low and high accumulation of antioxidants (unpublished). The phytochemical changes that occurred during maturation indicated that a significant difference was not only relative to tAsA and dAsA content, but also in their accumulation dynamic in fruit. Overall, AsA content positively correlated with the fruit stage, as in other pepper varieties as well as other horticultural species [34,35,36,37]. However, only for the higher accumulating variety PEP1, the major increment in AsA (47%, based on fresh weight) was produced during the transition from mature green to red ripe stage, a phase in which chloroplasts turn into chromoplasts. The lower accumulating PEP10 variety did not show a significant increase of AsA from mature green to red, which was not only of dietary relevance, but may affect the consumption and destination of use. Finally, in view of the limited difference in total polyphenols at fruit maturity, the data imply a higher nutritional value of PEP1, considering that the amount of vitamin C and polyphenols highly correlates with the antioxidant capacity in plants [1].

Differences in the level of AsA among pepper genotypes have been already reported by analysing fruits [34,38]. To understand if the observed variation is organ-specific, we quantified the tAsA, dAsA, and polyphenols in leaves. The higher accumulation in fruits and in leaves of PEP1 suggests that both constitutive (or at least organ-independent) and inducible mechanisms are responsible for different accumulation of the bioactive compounds. Moreover, the observed variability between the varieties also underlines the value of local pepper germplasm, which may also be relevant for other metabolic traits [38]. In the future, an analysis of a wider panel of varieties will have to confirm the predictive value of a comparative analysis of the AsA content in leaves, a strategy that can provide obvious advantages for screening large populations.

To shed light on the mechanisms involved in the regulation of AsA accumulation, we quantified the expression level of genes belonging to the pathways for AsA de novo synthesis, degradation and recycling. A more comprehensive understanding of all the genes involved in AsA metabolism could be addressed exploiting the power of NGS technologies. While the dynamic of the gene expression during maturation has been previously investigated [34,39], our aim was to compare behavior of the genes in the two contrasting varieties. Their relative gene expression analysis indicated that the increasing differences in AsA concentration during ripening are associated with a higher induction of genes involved in AsA biosynthesis. In comparison with leaves, additional pathways (e.g., myo-inositol and D-galacturonic acid) should contribute to the observed difference in sink tissues, as it was also observed for two tomato varieties with different levels of AsA in fruit [40]. In several studies, quantification of transcripts has been performed taking the earliest analysed stage as a reference. Under this perspective, the gene expression has a tendency to reduce towards maturation, while total ascorbate concentration increases. In this work, we calibrated the gene expression at each fruit stage, also to avoid possible confounding factors related to the variability in expression of housekeeping genes at different developmental and maturity stages. The comparative analysis implies that transcriptional regulation of the AsA biosynthesis, especially for genes of the L-galactose pathway, is a relevant factor for the differential accumulation in our pepper varieties [25,39,41]. GGP and GGP/VCT2 were overexpressed in PEP1 during the whole maturation process, consistent with their key role in regulating the AsA pool content reported in different plant species [22,41]. Similarly, MADHR1, whose involvement in AsA accumulation was studied in blueberry and tomato [42,43], was steadily upregulated in PEP1. The expression of all these genes significantly correlated with the AsA content in fruit. On the other hand, although AKR was also overexpressed in PEP1 during fruit development, its expression level did not correlate to tAsA content, which can be explained considering a genotype-specific contribution of the L-galactonic acid pathway to the AsA content of PEP1 fruits. The relative quantification also indicated that GME differential expression declined over maturation, being absent at the R stage. The expression of this gene weakly correlates with either tAsA or dAsA content. The GDP-D-mannose is a key enzyme connecting ascorbate synthesis to the primary cell wall metabolism in higher plants, and differences in the expression at IG and MG could be related to cell wall biosynthesis or more generally, to different fruit growth [44]. Accordingly, the expression of GME in leaves of the PEP10 was much lower than in PEP1 and not correlated with metabolites, consistent with a different tissue-specific regulation between varieties, as observed in celery [45].

The regulation of AsA concentrations during fruit ripening is a complex phenomenon that includes both transcriptional and post-transcriptional regulatory processes, also comprising feedback inhibition [22,25]. The higher expression of genes involved in ascorbic acid biosynthesis and regeneration is an important explanation for the observed higher AsA content in pepper fruits, as is also shown in other plant species for some genes. While transcript level is only one of the factors that modulate AsA abundance, by using a linear model approach, we found biologically meaningful gene-metabolite pairs that strongly correlated. The overexpression of the genes in the higher accumulating varieties and their clustering according to their correlation with tAsA also opens the possibility that the observed phenotype-specific relationships could rely on a single co-regulated molecular pattern [46]. The gene expression-metabolite correlations, although supported by the biochemical literature, do not necessarily imply causation, and further studies will have to strengthen the observed phenotype-gene relations in pepper and elucidate possible bio-modules of co-regulated genes.

## 4. Materials and Methods

### 4.1. Plant Material

This work was carried out on two locally cultivated varieties of *Capsicum annuum* L., “Puparuriello” (PEP1) and “Cazzone Rosso” (PEP10). Seeds were obtained from the breeding company “La Semiorto Sementi” (Salerno, Italy) and from the germplasm collection of the Dipartimento di Agraria, Sezione di Genetica e Biotecnologie Vegetali, Università degli Studi di Napoli “Federico II” (Naples, Italy), respectively. Seeds were germinated in modular seed trays and transplanted, approximately two weeks following germination, into 22 x 22 cm pots with a mixture (1:1) of volcanic (Vesuvian) sandy soil and commercial substrate (Type-S, FloraGard, Oldenburg, Germany). Plants were grown in greenhouse under natural light conditions at the Experimental Station of the Sezione di Genetica e Biotecnologie Vegetali, Università degli Studi di Napoli Federico II (Portici, NA), using standard cultural practices (e.g., watering and fertilization).

### 4.2. Metabolites Measurements

Total ascorbic acid, reduced ascorbic acid and polyphenol content was analysed in leaves and fruits. For the analysis of the leaf-content, leaves were collected from five plants at the second true leaf stage (approx. 40 days from transplant). For fruit analysis, three fruits per plants were harvested at the following stages: immature (IM), mature green (MG), and ripe-red (R) (Supplementary Materials, Figure S1), based on size and color. Analyses were carried out on three biological replicates. Leaves and fruit tissues, cut in pieces and separated from placenta and seeds, were stored at −80 °C until use. Frozen tissue (250 mg) was disrupted to a fine powder in a sterile ice-cold plastic tube with grinding beads (two 1 minute (min) bursts at 25 MHz), in 0.2 mL of 6% (*v*/*v*) of ice-cold trichloroacetic acid (TCA) using the TissueLyser system (Qiagen) in a cold room. Samples were then incubated 10 min in ice with occasional swirling. After centrifugation at 4 °C (14,000 rpm, 20 min), the clear supernatant was diluted to 0.5 mL with 6% TCA. Total ASA (tAsA) and reduced ascorbic acid (dAsA) were quantified, as previously described [47,48]. Briefly, an aliquot (50 µl) was added with 150 μL 0.2 M phosphate buffer (pH 7.4), 50 μL double distilled water, 250 μL 10% TCA, 200 μL 42% H_3_PO_4_, 200 μL 2,2′-dipyridyl (dpy), and 100 μL 3% FeCl_3_. An ethanol-no dpy sample was used to evaluate the contribution of interfering plant pigments. After a thorough mix, samples were incubated at 42 °C for 40 min before measuring light absorbance at 525 nm using a DU-640 UV/Vis spectrophotometer (Beckman Coulter). The AsA concentration was interpolated according to a reference calibration curve covering the 0–0.7 μmol AsA range. For polyphenol determination, frozen tissue (250 mg) was homogenized in 1 mL of 60% (*v*/*v*) MeOH with a TissueLyser (Qiagen) as above, and then samples were shaken for 30 min at 25 °C. After centrifugation (14,000 rpm, 15 min), the clear supernatant was diluted to 5 mL with 60% MeOH and then analyzed, as described [49]. Chemicals were purchased from Sigma-Aldrich.

### 4.3. Transcriptional Profiling

Total RNA isolation from leaves was performed as previously reported [47]. Total RNA from fruits was isolated using the RNeasy Plant Mini Kit from Qiagen, starting from around 200 mg of pulverised fruit tissue. RQ1 RNase-Free DNase (Promega) treatment, RNA quantification, synthesis of the first-strand cDNA, and Real Time Quantitative-PCR were carried out using already published procedures [47]. PCR reactions were assembled using the SYBR Green PCR Master Mix (Applied Biosystems) and run on an Applied Biosystems 7900 HT thermocycler. Reactions were performed in triplicates and quantification of gene expression was carried out using the ΔΔCt method [50]. Primers (purchased from Thermo Fisher Scientific) and PCR conditions were as described [34].

### 4.4. Statistical Analysis

For the metabolite measurements, significant differences between the two varieties were calculated using the independent samples *t*-tests, following testing for normality by the Levene’s test of homogeneity of variance. Values are expressed as the mean ± standard error of the mean (SE). For the gene expression analysis, statistical significance of the Relative Quantification (RQ) of gene expression between the two varieties was assessed evaluating whether the average 2^−ΔCt^ value of the test genotype was significantly different from that of the calibrator genotype (Student’s *t*-test). Analyses were performed with the SPSS 20 statistical package (IBM). For the integration of metabolic and gene expression data, we collectively used the measurements of the two varieties. For each fruit stage, gene expression data were normalized against the beta-tubulin expression level, averaged among the three replicates, and log_2_ transformed. tAsA and dAsA concentrations in replicated samples were first checked for consistency of abundance (i.e., coefficient of variation < 0.3) and then averaged. Integration of data followed a linear modelling approach (Pearson product-moment correlation coefficient). Clustering among genes was performed using the Average Linkage method (UPGMA) based on Euclidean distances. Calculations and graphical representation of specific gene-metabolite associations were carried out in R.

## Supplementary Materials

The following are available online at https://www.mdpi.com/2223-7747/8/7/206/s1, Figure S1: An example of the phenotype of the pepper fruits at the different harvesting times. **A**: PEP1 (immature green); **B**: PEP1 (mature green); **C**: PEP1 (red); **D**: PEP10 (immature green); **E**: PEP10 (mature green); and **F**: PEP10 (red). Scale bar: 3 cm. Figure S2: Ratio, expressed as percentage, between reduced ascorbic acid (dAsA) and total ascorbic acid (tAsA) concentration during fruit development. Data are mean ± s.d. of three biological replicates. IG: immature green; MG: mature green; R: red.

## References

[1] Martin C., Zhang Y., Tonelli C., Petroni K. (2013). Plants, diet, and health. Annu. Rev. Plant Biol..

[2] Liu R.H. (2003). Health benefits of fruit and vegetables are from additive and synergistic combinations of phytochemicals. Am. J. Clin. Nutr..

[3] Hossain M.A., Munné-Bosch S., Burritt D.J., Diaz-Vivancos P., Fujita M., Lorence A. (2017). Ascorbic Acid in Plant. Growth, Development and Stress Tolerance.

[4] Wildman R.E.C. (2016). Handbook of Nutraceuticals and Functional Foods.

[5] Foyer C.H. (2017). Ascorbic Acid. Antioxidants in Higher Plants.

[6] Pandey K.B., Rizvi S.I. (2009). Current understanding of dietary polyphenols and their role in health and disease. Curr. Nutr. Food Sci..

[7] Nicholson R.L., Hammerschmidt R. (1992). Phenolic compounds and their role in disease resistance. Annu. Rev. Phytopathol..

[8] Tomás-Barberán F.A., Espín J.C. (2001). Phenolic compounds and related enzymes as determinants of quality in fruits and vegetables. J. Sci. Food Agric..

[9] Tavarini S., Degl’Innocenti E., Remorini D., Massai R., Guidi L. (2008). Antioxidant capacity, ascorbic acid, total phenols and carotenoids changes during harvest and after storage of Hayward kiwifruit. Food Chem..

[10] Ghasemnezhad M., Sherafati M., Payvast G.A. (2011). Variation in phenolic compounds, ascorbic acid and antioxidant activity of five coloured bell pepper (*Capsicum annum*) fruits at two different harvest times. J. Funct. Foods.

[11] Cruz-Rus E., Botella M.A., Valpuesta V., Gomez-Jimenez M.C. (2010). Analysis of genes involved in L-ascorbic acid biosynthesis during growth and ripening of grape berries. J. Plant. Physiol..

[12] Topuz A., Ozdemir F. (2007). Assessment of carotenoids, capsaicinoids and ascorbic acid composition of some selected pepper cultivars (*Capsicum annuum* L.) grown in Turkey. J. Food Compos. Anal..

[13] Deepa N., Kaur C., George B., Singh B., Kapoor H. (2007). Antioxidant constituents in some sweet pepper (*Capsicum annuum* L.) genotypes during maturity. LWT Food Sci. Technol..

[14] Davey M.W., van Montagu M., Inze D., Sanmartin M., Kanellis A., Smirnoff N., Benzie I.J.J., Strain J.J., Favell D., Fletcher J. (2000). Plant L-ascorbic acid: Chemistry, function, metabolism, bioavailability and effects of processing. J. Sci. Food Agric..

[15] Valpuesta V., Botella M.A. (2004). Biosynthesis of L-ascorbic acid in plants: New pathways for an old antioxidant. Trends Plant Sci..

[16] Conklin P. (2001). Recent advances in the role and biosynthesis of ascorbic acid in plants. Plant Cell Environ..

[17] Smirnoff N. (2018). Ascorbic acid metabolism and functions: A comparison of plants and mammals. Free Radic. Biol. Med..

[18] Horemans N., Foyer C.H., Asard H. (2000). Transport and action of ascorbate at the plant plasma membrane. Trends Plant Sci..

[19] Wheeler G.L., Jones M.A., Smirnoff N. (1998). The biosynthetic pathway of vitamin C in higher plants. Nature.

[20] Wheeler G., Ishikawa T., Pornsaksit V., Smirnoff N. (2015). Evolution of alternative biosynthetic pathways for vitamin C following plastid acquisition in photosynthetic eukaryotes. Elife.

[21] Chen Z., Young T.E., Ling J., Chang S.C., Gallie D.R. (2003). Increasing vitamin C content of plants through enhanced ascorbate recycling. Proc. Natl. Acad. Sci. USA.

[22] Mellidou I., Kanellis A.K. (2017). Genetic control of ascorbic acid biosynthesis and recycling in horticultural crops. Front. Chem..

[23] Smirnoff N., Wheeler G.L. (2000). Ascorbic acid in plants: Biosynthesis and function. Crit. Rev. Plant Sci..

[24] Dowdle J., Ishikawa T., Gatzek S., Rolinski S., Smirnoff N. (2007). Two genes in *Arabidopsis thaliana* encoding GDP-l-galactose phosphorylase are required for ascorbate biosynthesis and seedling viability. Plant J..

[25] Bulley S., Laing W. (2016). The regulation of ascorbate biosynthesis. Curr. Opin. Plant Biol..

[26] Murgia I., De Gara L., Grusak M.A. (2013). Biofortification: How can we exploit plant science and biotechnology to reduce micronutrient deficiencies?. Front. Plant Sci..

[27] Li H., Huang W., Wang G.L., Wang W.L., Cui X., Zhuang J. (2017). Transcriptomic analysis of the biosynthesis, recycling, and distribution of ascorbic acid during leaf development in tea plant (*Camellia sinensis* (L.) O. Kuntze). Sci. Rep..

[28] George G.M., Ruckle M.E., Abt M.R., Bull S.E. (2017). Ascorbic Acid Biofortification in Crops. Ascorbic Acid in Plant Growth, Development and Stress Tolerance.

[29] Locato V., Cimini S., De Gara L. (2013). Strategies to increase vitamin C in plants: From plant defense perspective to food biofortification. Front. Plant Sci..

[30] Haroldsen V.M., Chi-Ham C.L., Kulkarni S., Lorence A., Bennett A.B. (2011). Constitutively expressed DHAR and MDHAR influence fruit, but not foliar ascorbate levels in tomato. Plant Physiol. Biochem..

[31] Wanichthanarak K., Fahrmann J.F., Grapov D. (2015). Genomic, proteomic, and metabolomic data integration strategies. Biomark. Insights.

[32] Macknight R.C., Laing W.A., Bulley S.M., Broad R.C., Johnson A.A., Hellens R.P. (2017). Increasing ascorbate levels in crops to enhance human nutrition and plant abiotic stress tolerance. Curr. Opin. Biotechnol..

[33] Della Penna D. (1999). Nutritional genomics: Manipulating plant micronutrients to improve human health. Science.

[34] Alós E., Rodrigo M.J., Zacarías L. (2013). Transcriptomic analysis of genes involved in the biosynthesis, recycling and degradation of L-ascorbic acid in pepper fruits (*Capsicum annuum* L.). Plant Sci..

[35] Ioannidi E., Kalamaki M.S., Engineer C., Pateraki I., Alexandrou D., Mellidou I., Giovannonni J., Kanellis A.K. (2009). Expression profiling of ascorbic acid-related genes during tomato fruit development and ripening and in response to stress conditions. J. Exp. Bot..

[36] Martínez S., López M., González-Raurich M., Bernardo Alvarez A. (2005). The effects of ripening stage and processing systems on vitamin C content in sweet peppers (*Capsicum annuum* L.). Int. J Food Sci. Nutr..

[37] Yahia E.M., Contreras-Padilla M., Gonzalez-Aguilar G. (2001). Ascorbic acid content in relation to ascorbic acid oxidase activity and polyamine content in tomato and bell pepper fruits during development, maturation and senescence. LWT Food Sci. Technol..

[38] Wahyuni Y., Ballester A.R., Sudarmonowati E., Bino R.J., Bovy A.G. (2011). Metabolite biodiversity in pepper (*Capsicum*) fruits of thirty-two diverse accessions: Variation in health-related compounds and implications for breeding. Phytochemistry.

[39] Del Rocío Gómez-García M., Ochoa-Alejo N. (2016). Predominant role of the l-galactose pathway in l-ascorbic acid biosynthesis in fruits and leaves of the *Capsicum annuum* L. chili pepper. Braz. J. Bot..

[40] Mellidou I., Keulemans J., Kanellis A.K., Davey M.W. (2012). Regulation of fruit ascorbic acid concentrations during ripening in high and low vitamin C tomato cultivars. BMC Plant Biol..

[41] Li M., Ma F., Liang D., Li J., Wang Y. (2010). Ascorbate biosynthesis during early fruit development is the main reason for its accumulation in kiwi. PLoS ONE.

[42] Liu F., Wang L., Gu L., Zhao W., Su H., Cheng X. (2015). Higher transcription levels in ascorbic acid biosynthetic and recycling genes were associated with higher ascorbic acid accumulation in blueberry. Food Chem..

[43] Sauvage C., Segura V., Bauchet G., Stevens R., Do P.T., Nikoloski Z., Fernie A.R., Causse M. (2014). Genome Wide Association in tomato reveals 44 candidate loci for fruit metabolic traits. Plant Physiol..

[44] Mounet-Gilbert L., Dumont M., Ferrand C., Bournonville C., Monier A., Jorly J., Lemaire-ChamLey M., Mori K., Atienza I., Hernould M. (2016). Two tomato GDP-D-mannose epimerase isoforms involved in ascorbate biosynthesis play specific roles in cell wall biosynthesis and development. J. Exp. Bot..

[45] Huang W., Wang G.L., Li H., Wang F., Xu Z.S., Xiong A.S. (2016). Transcriptional profiling of genes involved in ascorbic acid biosynthesis, recycling, and degradation during three leaf developmental stages in celery. Mol. Genet. Genom..

[46] Gaudinier A., Tang M., Kliebenstein D.J. (2015). Transcriptional networks governing plant metabolism. Curr. Plant Biol..

[47] Zarrillo A., Minutolo M., Alioto D., Errico A. (2017). Ascorbic acid regulation in leaves and fruits of tomato ecotypes infected by Eggplant Mottled Dwarf Virus. Sci. Hortic..

[48] Kampfenkel K., Van Montagu M., Inzé D. (1995). Effects of iron excess on *Nicotiana plumbaginifolia* plants (implications to oxidative stress). Plant Physiol..

[49] Minutolo M., Amalfitano C., Evidente A., Frusciante L., Errico A. (2013). Polyphenol distribution in plant organs of tomato introgression lines. Nat. Prod. Res..

[50] Livak K.J., Schmittgen T.D. (2001). Analysis of relative gene expression data using real-time quantitative PCR and the 2−ΔΔCT method. Methods.

