# Supplementary figures and images for: Transcriptional Regulation of Ascorbic Acid During Fruit Ripening in Pepper (Capsicum annuum) Varieties with Low and High Antioxidants Content
Source: Plants (Basel). 2019 Jul 4;8(7):206. doi: 10.3390/plants8070206 (PMC6681188; doi:10.3390/plants8070206)

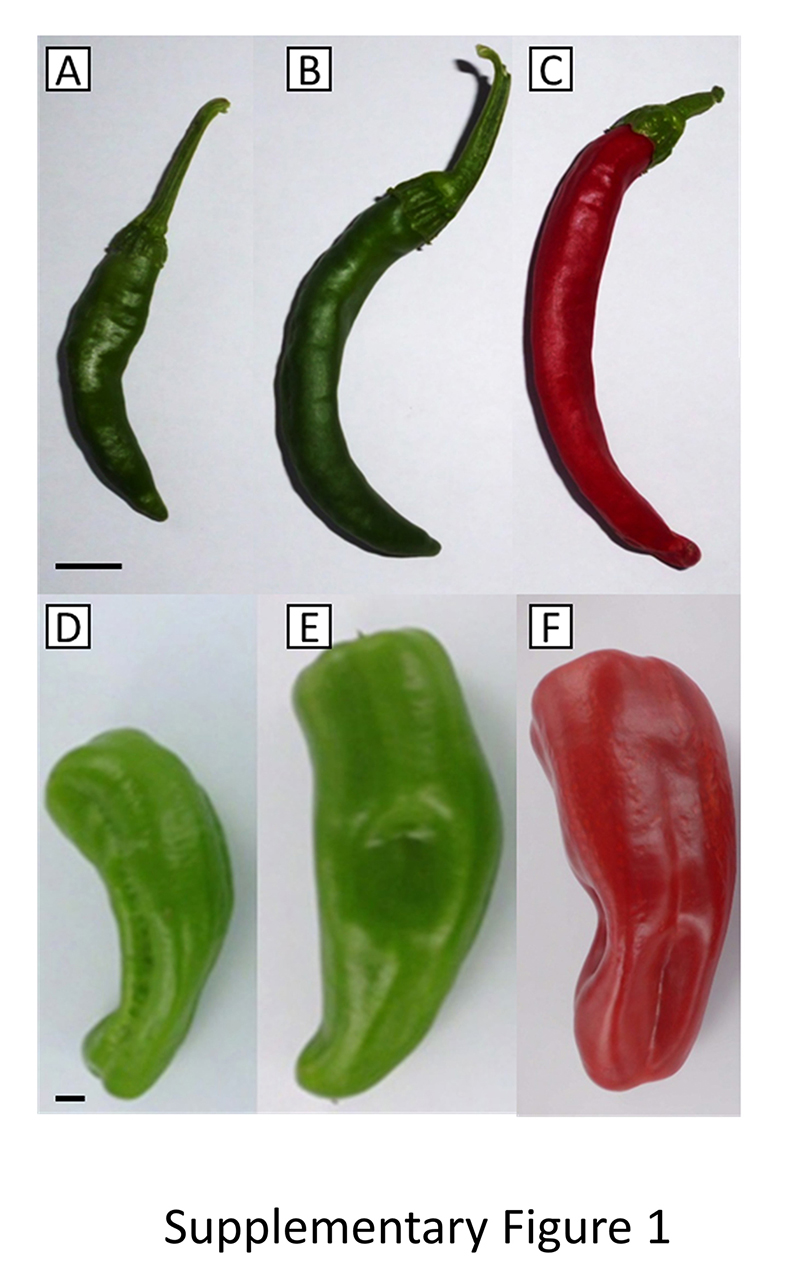

Supplement: Supplementary file 1 [file plants-08-00206-s001.zip › Supplementary Figure 1.jpg]

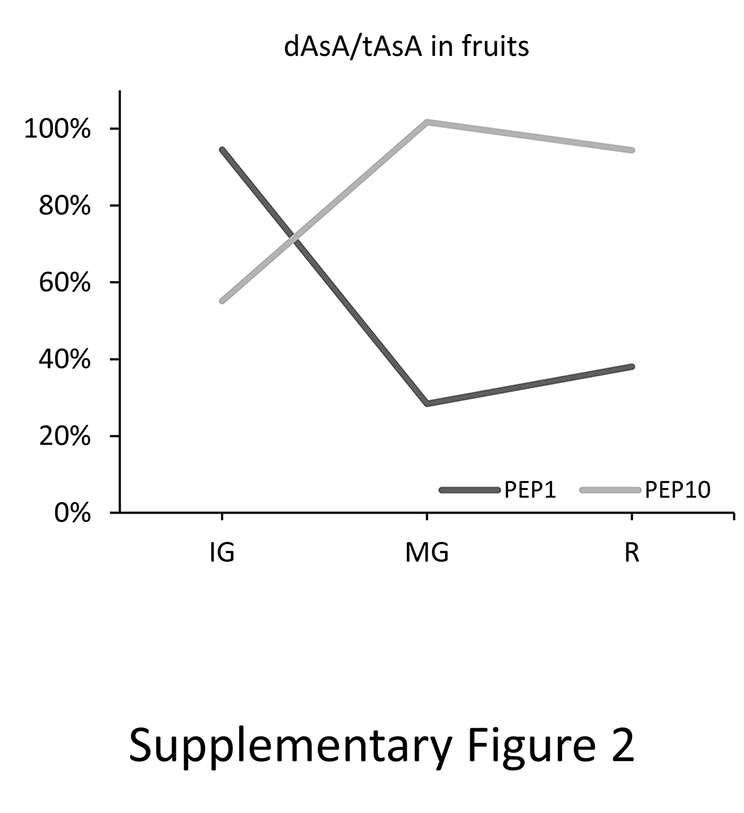

Supplement: Supplementary file 1 [file plants-08-00206-s001.zip › Supplementary Figure 2.jpg]
